# Ultranarrow Germanene Nanoribbons with Pentagonal Rings

**DOI:** 10.1021/acs.nanolett.6c02715

**Published:** 2026-07-22

**Authors:** Kewei Sun, Orlando J. Silveira, Adam S. Foster, Shigeki Kawai

**Affiliations:** † Center for Basic Research on Materials, National Institute for Materials Science, 1-2-1 Sengen, Tsukuba, Ibaraki 305-0047, Japan; ‡ International Center for Young Scientists, National Institute for Materials Science, 1-2-1 Sengen, Tsukuba, Ibaraki 305-0047, Japan; § Vacuum Interconnected Nanotech Workstation, Suzhou Institute of Nano-Tech and Nano-Bionics, 603032 Chinese Academy of Sciences, Suzhou, 215123, China; ∥ Department of Applied Physics, 174277 Aalto University, Espoo 02150, Finland; ⊥ Nano Life Science Institute (WPI-NanoLSI), Kanazawa University, Kakuma-machi 920-1192, Japan; # Graduate School of Pure and Applied Sciences, University of Tsukuba, Tsukuba 305-8571, Japan

**Keywords:** germanene nanoribbons, pentagonal rings, scanning tunneling microscopy/spectroscopy, density functional theory, field-effect resonant tunneling spectroscopy

## Abstract

One-dimensional elemental nanostructures, exemplified by graphene nanoribbons, hold great potential for advanced nanoelectronics, which can be further expanded by composition of heavier group 14 elements. Although the synthesis of germanene nanoribbons (GeNRs) has been recently demonstrated through high-temperature Ge atom segregation onto Pt and Ag adlayers from stepped Ge(110) bulk crystals, atomic-scale control of their structures remains unexplored. Here, we present ultranarrow GeNRs composed exclusively of pentagonal rings via thermal treatment of germanium clusters onto Ag(111) at 300 K with high-resolution scanning tunneling microscopy. Field-effect resonant tunneling spectra supported by *ab initio* calculations reveal that the work function of GeNRs is moderately higher than that of pristine Ag(111), resulting in a directional interfacial charge modulation. Our findings suggest that the GeNR/Ag(111) system offers an atomically defined platform for studying metal contacts, work function engineering, resonant tunneling, and substrate-stabilized one-dimensional electronic states as well as mechanical properties.

## Graphical abstract

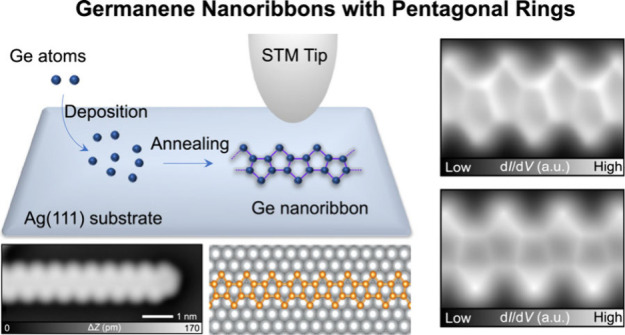

Quasi one-dimensional (1D) nanomaterials are viewed as key functional materials for nanoelectronic applications, offering promising opportunities for the design and fabrication of nanoscale devices.^1–5^ Representative examples include transition-metal chalcogenide nanowires (MoS_2_, MoSe_2_, MoTe_2_),^1^ oxide nanowires (ZnO, TiO_2_),^2^ carbon nanotubes (CNTs),^3^ and π-conjugated polymers.^4^ Among them, atomically precise single-element nanoribbons have attracted significant attention due to the compositional simplicity, making them ideal model systems for investigating various physical and chemical properties as well as tailored applications. A prominent example is graphene nanoribbons (GNRs), which are typically synthesized via the on-surface polymerization of small organic molecules.^6–14^ In contrast, silicene nanoribbons (SiNRs),^15–18^ phosphorene nanoribbons (PNRs)^19–21^ and borophene nanoribbons (BNRs)^22–24^ have been successfully fabricated by directly depositing Si, P, or B atoms onto suitable substrates, respectively. Recently, germanene nanoribbons (GeNRs) have been formed via segregation on a Pt or Ag monolayer on Ge(110) substrates.^25,26^ GeNRs prepared by this method tend to exhibit nonuniform widths due to the highly corrugated structure of the substrate. In addition, such GeNRs were attributed to consist of hexagonal rings, with a structure similar to that of zigzag-type GNRs. However, atomically controlled narrow GeNRs composed of pentagonal rings^27^ have not yet been realized.

Here, we present the synthesis of ultranarrow GeNRs composed of pentagonal rings on Ag(111). Ge atoms were deposited on a silver substrate that was kept at 150 K. Upon subsequent annealing to room temperature, nanoribbons grew from the edges of Ge clusters along the ⟨110⟩ direction. Bond-resolved scanning tunneling microscopy (STM) combined with density functional theory (DFT) calculations revealed that the GeNRs consist of only repeating pentagonal rings with alternating orientations. The metallic character of the GeNR on Ag(111) was identified by scanning tunneling spectroscopy (STS) measurements. This property arises from electronic coupling with the substrate and departs from conventional sp^2^ and sp^3^ hybridizations, as further confirmed by DFT calculations. Field-effect resonant tunneling (FERT) spectroscopy measurements revealed the local work function of GeNRs to be approximately 4.87 eV, slightly higher than that of the Ag substrate, leading to a directional interfacial charge transfer. The GeNRs can be bent by the STM tip, demonstrating great potential as 1D flexible inorganic nanomaterials. Our findings provide a foundation for the future fabrication and application of designer-GeNR-based devices.

Germanium atoms were deposited on the Ag(111) surface kept at 150 K (Figure S1), followed by annealing to room temperature (approximately 300 K) for 1 h. The sample was subsequently cooled to 4.3 K for STM characterization. A schematic illustration of the preparation and characterization processes is shown in Figure 1a. Uniform nanoribbon structures emerged from the edges of Ge clusters (Figure 1b), with their longitudinal axes aligned along the <110> lattice direction of the silver surface. A close-up view of the individual nanoribbon (Figure 1c) shows serrated bright spots distributed along its edges. Figures 1d and 1e show the top and side view of a proposed structure of the uniform Ge nanoribbon relaxed with DFT, which is composed of repeating pentagonal rings oriented in opposite directions that are compatible with the serrated bright spots seen in Figure 1c. The atomic arrangement of the nanoribbon on the substrate was obtained by structural optimization using a periodic ribbon–substrate model. The DFT relaxation shows that the proposed Ge nanoribbon remains stable and planar atop the surface. The proposed structure was then experimentally confirmed by acquiring a series of constant height d*I*/d*V* maps with a CO-terminated tip apex, resolving their inner structures.^28,29^ The d*I*/d*V* maps in Figure 1f demonstrate that the nanoribbons are indeed composed of intercalating Ge pentagons, even at the ribbon terminus (Figure 1h). Figure 1g shows DFT-simulated d*I*/d*V* maps considering a relaxed CO tip. The resemblance between the experiment and simulations fully confirms the demonstration of a Ge nanoribbon consisting of pentagonal rings, which, to the best of our knowledge, have not yet been reported. In previous studies, the GeNRs formed on Ag(110)/Ge(110) appeared to adopt a honeycomb lattice structure.^25,26^ In addition, direct deposition of Ge atoms onto Ag(110) under suitable conditions produces an ordered superstructure composed of two strongly buckled Ge tetramer units.^30^ By contrast, direct deposition of Ge onto Al(110) yields one-dimensional atomic chain structures. However, their atomic structure remains unresolved and has been proposed to consist of either six-membered rings or nonplanar 10-membered rings.^31^ While GeNRs are generally made of hexagonal rings, the possible presence of pentagonal rings has not yet been clarified. Interestingly, similar pentagonal ring structures have also been observed in SiNRs,^18,19^ suggesting that such configurations may arise in ultranarrow nanoribbons composed of heavier than carbon group IVA elements. The recorded lattice parameter of the GeNR is 0.58 ± 0.1 nm (indicated by an arrow in Figure 1c), which agrees well with the calculated value of 0.578 nm. The measured apparent height of the GeNR is about 120 pm relative to the Ag(111) substrate (Figure 1i). In addition, we attempted to obtain optimized growth parameters by tuning the substrate temperature during germanium deposition (Figure S2). When Ge atoms are deposited at a low temperature (e.g., 150 K), surface diffusion is suppressed, so they remain as clusters. During subsequent RT annealing, thermal energy enables Ge atoms to rearrange in an ordered way, facilitating the formation of GeNRs. We also found that GeNRs become unstable above 320 K (Figure S3), explaining why previous high-temperature annealing methods failed to produce such pentagonal ring-based GeNRs.

**Figure 1:**
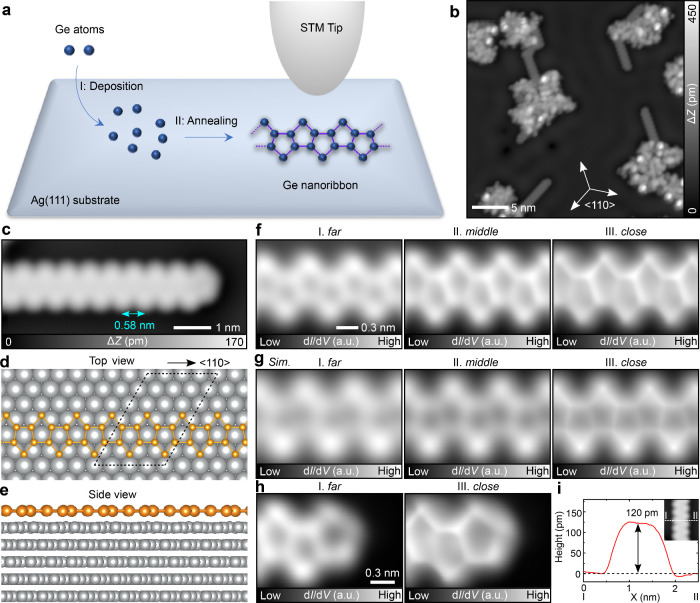
Fabrication of germanene nanoribbons (GeNRs) on Ag(111). (**a**) Scheme of fabrication of GeNRs on Ag(111) and characterization by STM. (**b**) Large-scale STM topography of the Ag(111) surface after annealing at room temperature for 1 h. (**c**) Close-up view of the individual GeNR. (**d**) Top and (**e**) side view of the optimized GeNR on top of 5 layers of Ag(111). The dashed line in (d) corresponds to the unit cell of the GeNR/Ag(111) structure, with lattice parameter 11.56 Å (twice the lattice parameter of the GeNR, commensurate with the underlying Ag(111) substrate). (**f**) Constant height d*I*/d*V* maps of GeNR segment recorded with a CO-tip at sample bias *V* = 1 mV and modulation voltage *V*_ac_ = 10 mV, acquired at far, middle, and close tip–surface distances. (**g**) Simulated d*I*/d*V* maps of a GeNR segment considering a relaxed CO-terminated tip. The simulations were acquired considering three distances between the O atom and the GeNR: 5.1 Å (far), 4.1 Å (middle), and 3.6 Å (close). (**h**) Constant height d*I*/d*V* maps of the GeNR terminal recorded with a CO-tip at *V* = 1 mV and *V*_ac_ = 10 mV (far and close). (**i**) Line profile taken along line I–II in the inset image. Measurement parameters: sample bias voltage *V* = 100 mV and tunneling current *I* = 10 pA in (b), *V* = 200 mV and *I* = 5 pA in (c).

Next, we investigate the electronic properties of the GeNR by STS. d*I*/d*V* spectra (Figure 2a) were acquired at the sites on the GeNR indicated by colored dots in the inset of Figure 2a, as well as on the bare Ag(111) surface (gray curve) for reference. The measured spectra show a small broad peak near zero bias, implying that the nanoribbon becomes metallic. A similar situation is observed for silicon nanoribbons with pentagonal rings on Ag(110), which also exhibit metallic behavior.^17^ Figure 2b shows the DFT-calculated projected density of states (PDOS) on selected Ge atoms, as well as on their perpendicular (p_*z*_) and parallel (p_*x*_+p_*y*_) orbitals. The inset in Figure 2b shows schematically the two- and three-coordinated Ge atoms that compose the GeNR, with the two-coordinated Ge lying at the edge of the ribbon, and the color scale indicates the Bader charge of each atom.^32–35^ The two-coordinated Ge atoms tend to lose electrons (0.15e), while the three-coordinated ones tend to either be neutral or gain electrons (up to 0.03e). Overall, the PDOS on the p_*z*_ orbitals of both two- and three-coordinated Ge atoms is broadly distributed over the energy range of −2 to 2 eV, without pronounced features at the Fermi level. The behavior of the p_*z*_ orbitals in the PDOS is compatible with the broad features in the experimental d*I*/d*V*, as it probes the local density of states with significant out-of-plane character. On the other hand, the planar p_*x*_+p_*y*_ orbitals are quite anisotropic in relation to p_*z*_, with relevant contribution for energies above 0.5 eV and below −0.5 eV that indicate their role in σ bonding within the GeNR. This strong anisotropy between planar and perpendicular orbitals, together with the largely planar geometry of the nanoribbon, rules out sp^3^ hybridization. At the same time, the absence of distinct π features given by the broad behavior of the p_*z*_ orbitals rules out sp^2^ hybridization as well, suggesting that the substrate interaction dominates the hybridization scheme. The Ag(111) substrate readily forms strong hybridization with epitaxially grown nanostructures, such as silicene^36^ and germanene.^37^ Indeed, the charge difference isosurface shown in Figure 2c displays a complex charge redistribution. Electrons accumulate at the interface, which are shared in the bonding region between both the GeNR and the Ag surface. Because of that, within each Ge atom, electrons are pulled upward, away from the surface, creating a dipole in each atom. This picture becomes clearer in the planar average charge density difference plot (Figure 2d), where spikes are seen near the GeNR/Ag(111) interface.

**Figure 2:**
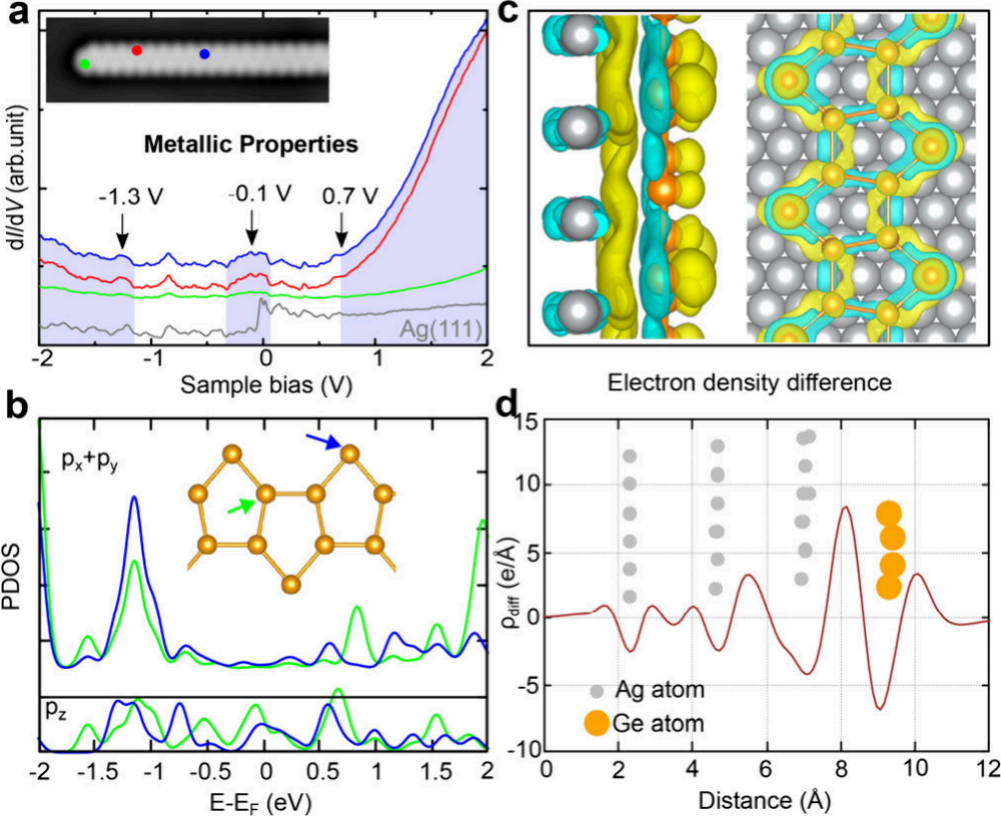
Electronic properties of GeNR on Ag(111). (**a**) d*I*/d*V* spectra recorded at different sites over the GeNR (indicated by color dots in the inset) and the bare Ag(111) surface. (**b**) Calculated projected density of states (PDOS); the inset shows a portion of the GeNR structure. Blue curves correspond to edge Ge atoms (marked by blue arrow in the inset), while green curves represent inner Ge atoms within the nanoribbon (marked by a green arrow in the inset). (**c**) Side and top view of the DFT-calculated electron density difference (±0.003 charge contours). Yellow isosurface reflects charge accumulation, while blue reflects depletion. (**d**) Planar average charge density difference plot. The small circular drawing shows the side view of the structure: orange circles are Ge atoms and gray circles are Ag atoms.

Owing to the strong hybridization between the GeNR and the substrate, the measured d*I*/d*V* spectra reflect the apparent electronic states of the GeNR/substrate system rather than the intrinsic electronic structure of the freestanding ribbon. Further apparent electronic states are observed in the occupied region at approximately −1.3 V (Figure 2a), whereas the unoccupied states begin to appear at around 0.7 V without a distinct spectral peak. To examine their spatial distribution, constant current d*I*/d *V* maps were acquired at representative bias voltages (Figure S4). The occupied state at −1.3 V is mainly localized at the ribbon edges. The d*I*/d*V* maps recorded at 0.7, 0.9, 1.1, and 1.3 V exhibit similar edge-localized contrast, indicating that the unoccupied states evolved from the onset at 0.7 V. In addition, standing waves are observed around the GeNR (Figure S4) at these bias voltages, further revealing electronic coupling between the GeNR and the substrate.^38^ In contrast, the state near the Fermi level (−0.1 V) extends across both the edges and the central region of the GeNR.

To determine the local work function of the GeNRs on Ag(111), we performed field-effect resonant tunneling (FERT) spectroscopy measurements. The corresponding image potential states (IPS)^39^ can be detected between the tip and sample surface (Figure 3a). In the quasi-classical approximation, resonant tunneling takes place when the Fermi level of the tip aligns with a Stark-shifted IPS:^40,41^

$$eV_{n}=Ø+{\left(\frac{3n\pi \hbar eE}{\sqrt{2m}}\right)}^{2/3}$$ 1

Here, *V*_*n*_ refers to the sample voltage corresponding to the *n*th resonance, ϕ the local work function of the sample surface, *m* the free electron mass, and *E* the applied electric field. FERT spectra (represented as d*Z*/d*V* curves) in blue and black (Figure 3b) were obtained by sweeping the sample bias at a constant current of 3 pA on GeNR and Ag(111), respectively. The IPS peaks at *n* = 1, 2, 3, ..., on GeNR are shifted to higher energies, indicating a higher local work function compared to the Ag(111) substrate. Such an energy shift is also observed in the two-dimensional d*Z*/d*V* map (Figure 3d), which is derived from 121 spectra taken along the line I–II (inset of Figure 3b). The energy positions e*V*_*n*_ of the IPS states were plotted as a function of *n* ^2/3^, as illustrated in Figure 3c. Fitting the data with eq 1 (Figure 3c) yielded a work function of 4.53 eV for the Ag(111) substrate, in agreement with previous reports.^42^ The local work function of GeNR was determined to be 4.87 eV, which is 0.34 eV higher than that of Ag(111). These findings suggest electron transfer from the Ag substrate to the GeNRs.^20,41,43^ DFT calculations indicate a slight increase of the work function of 0.10 eV (from 4.35 eV for bare Ag to 4.45 eV for GeNR+Ag), smaller than the experimental value (as generally expected^44^) and in agreement with previous calculations^45^ but following the same trend of charge transfer. Indeed, the Bader charge values show charge transfer from the Ag substrate to the three-coordinated Ge atoms, which are present within the ribbon.

**Figure 3:**
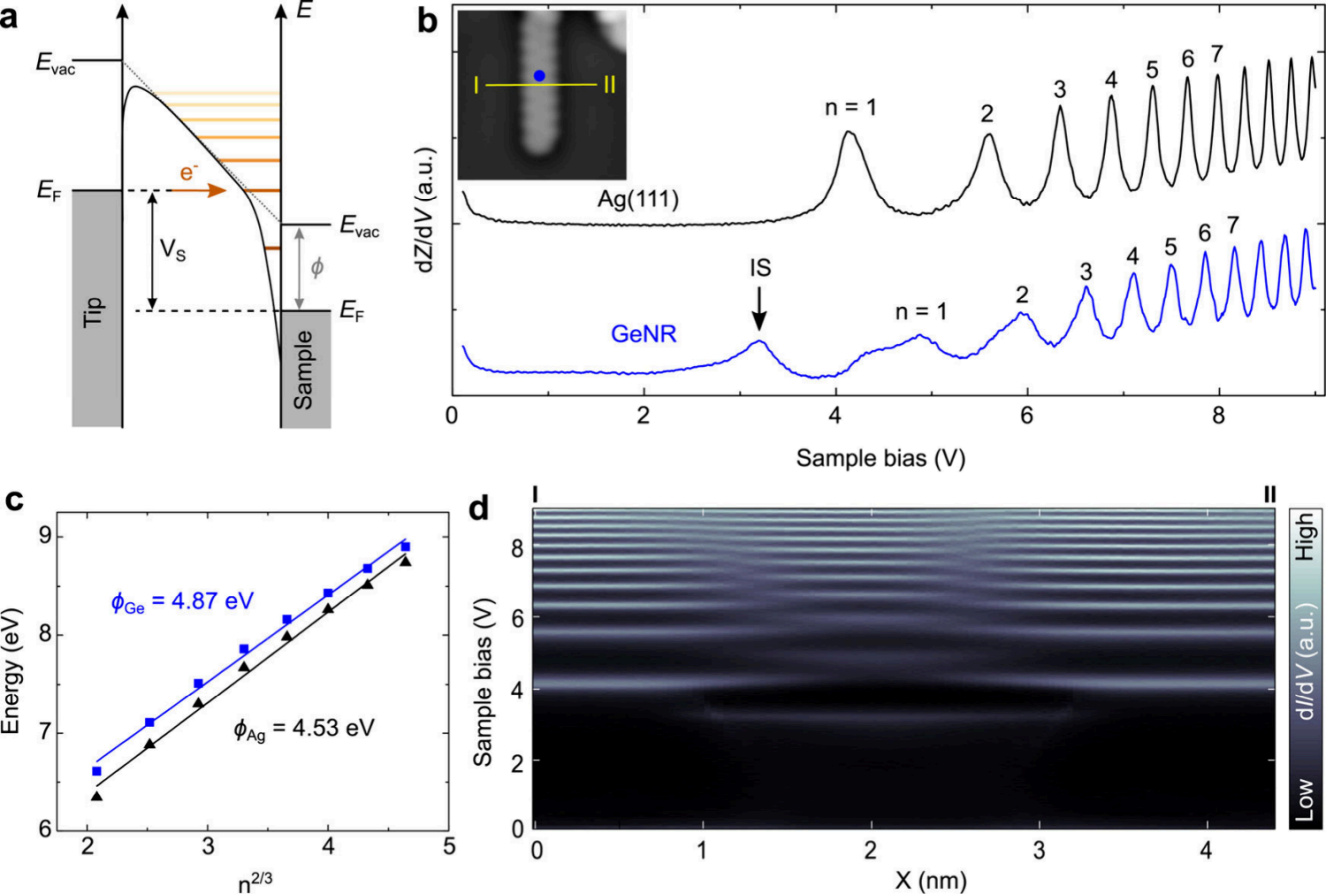
Work function of the GeNR on Ag(111). (**a**) Illustration of the band alignment under the Fowler–Nordheim tunneling regime with the Stark-shifted IPS. (**b**) FERT spectra were recorded at sites above the GeNR and the bare Ag(111) surface. (**c**) Extracted and fitted IPS peak positions. (**d**) FERT spectra were acquired across the GeNR along the line I–II shown in panel (b).

To gain insight into the mechanical properties of GeNRs, we conducted tip-induced manipulation experiments. As a first step, we aimed to assess the strength of the Ge–Ge bonds in a single GeNR on Ag(111). Upon moving the tip (as indicated by the arrows in Figure 4a), the GeNR was cut into two segments (Figure 4b). Such cutting was not achieved in GNRs on metal surfaces, indicating that the Ge–Ge bond is weaker than the C–C bond, as expected.^46^ Next, we dragged the terminus of a straight GeNR using tip manipulation (Figure 4c, Figure S5). The GeNR became bent during the process but remained unbroken (Figure 4d), indicating that the Ge–Ge bond is stronger than the interaction between Ge atoms and the underlying Ag substrate. After bending, most pentagonal rings of the GeNR are still resolved in high-resolution images (Figure S6), suggesting that the overall structure of the nanoribbon is not significantly disrupted. This also demonstrates the notable mechanical flexibility of GeNRs, making them promising 1D flexible nanomaterials. The DFT-calculated absorption energy of the GeNR on Ag(111) is 102 meV/atom, whereas the absorption energy of a single Ge atom at the hollow site of Ag(111) is 34 meV/atom, indicating that the GeNR is clearly more favorable in energy.

**Figure 4:**
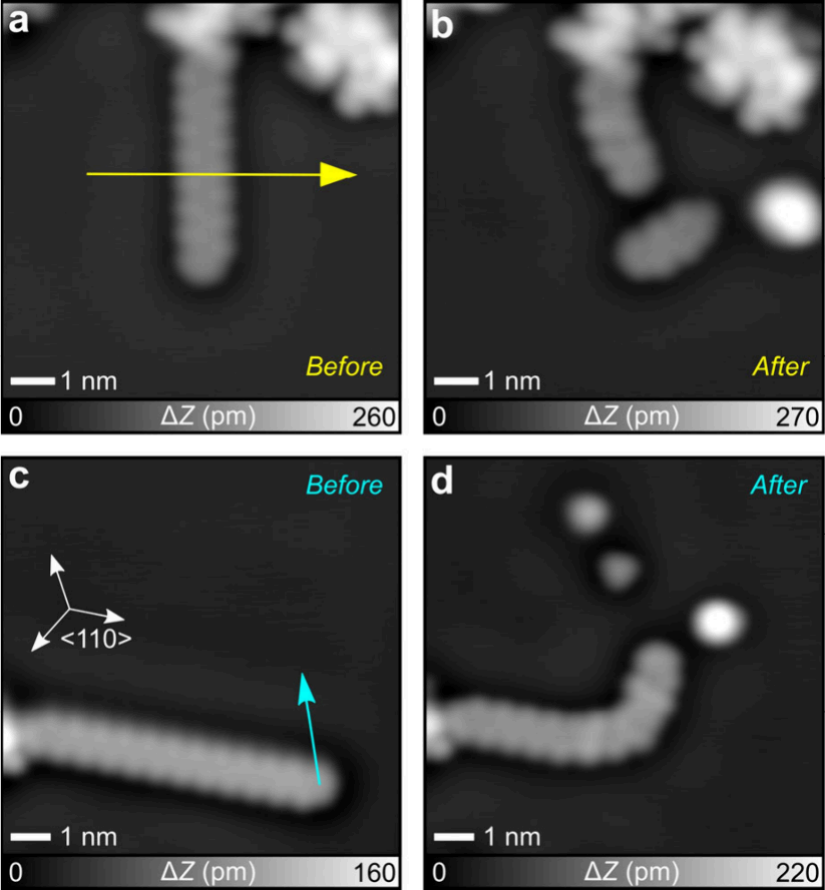
Tip-induced manipulation of GeNRs on Ag(111). Tip-assisted cutting of a single GeNR: (**a**) before and (**b**) after the manipulation. Tip-assisted lateral movement of an individual GeNR: (**c**) before and (**d**) after the manipulation. Arrows indicate the direction of tip movement. Measurement parameters: *V* = 200 mV and *I* = 5 pA in (a) and (b). *V* = 200 mV and *I* = 10 pA in (c) and (d).

In this study, we present a straightforward and effective strategy for the fabrication of GeNRs comprising only pentagonal rings. Ge atoms deposited on the Ag(111) surface at low temperature and subsequently annealed at room temperature yield GeNRs. Bond-resolved STM characterization shows that these GeNRs are composed of side-by-side pentagonal rings, further supported by DFT calculations. The GeNR displays metallic character on Ag(111), originating from electronic hybridization with the underlying substrate. Moreover, the nanoribbon exhibits a local work function slightly higher than that of the pristine Ag(111) surface, inducing interfacial charge redistribution and polarization. STM tip manipulation verifies the Ge–Ge bonds possess favorable flexibility, enabling the material to serve as 1D flexible inorganic nanostructures. These results provide important insights into the properties of pentagonal-ring Ge nanoribbons and establish a foundation for their future application in nanoelectronic devices.

## Supplementary Material



## Notes

Experimental and theoretical methods, as-deposited Ge atoms on Ag(111) held at 150 K, growth of GeNRs on Ag(111), thermal stability test at 320 K, electronic properties of GeNR on Ag(111), tip-induced manipulation of GeNRs on Ag(111), electronic properties of GeNR on Ag(111) calculated with the PBE functional, Bader charges of the inequivalent Ge atoms in the GeNR (PDF)
