# Supplementary material for: Ultranarrow Germanene Nanoribbons with Pentagonal Rings
Source: Nano Lett. 2026 Jul 22;26(30):9976–81. doi: 10.1021/acs.nanolett.6c02715 (PMC13450475; doi:10.1021/acs.nanolett.6c02715)
Supplement: Supplementary file 1 [file nl6c02715_si_001.pdf]

Supplementary Materials for
Ultra-Narrow Germanene Nanoribbons with Pentagonal Rings

Kewei Sun[1,2,3]*, Orlando J. Silveira[4], Adam S. Foster[4,5]*, Shigeki Kawai[1,6]*

[1]*Center for Basic Research on Materials, National Institute for Materials Science, 1-2-1 Sengen, Tsukuba, Ibaraki 305-0047, Japan.*

[2]*International Center for Young Scientists, National Institute for Materials Science, 1-2-1 Sengen, Tsukuba, Ibaraki 305-0047, Japan.*

[3]*Vacuum Interconnected Nanotech Workstation, Suzhou Institute of Nano-Tech and Nano-Bionics, Chinese Academy of Sciences, Suzhou 215123, China.*

[4]*Department of Applied Physics, Aalto University, Espoo 00076, Finland.*

[5]*Nano Life Science Institute (WPI-NanoLSI), Kanazawa University, Kakuma-machi, Kanazawa 920-1192, Japan.*

[6]*Graduate School of Pure and Applied Sciences, University of Tsukuba, Tsukuba 305-8571, Japan.*

Experimental and theoretical methods

STM experiments: All experiments were conducted using a home-made low temperature scanning tunneling microscopy (STM) system at 4.3 K under ultrahigh vacuum condition (< 5×10^{-10} mbar). A clean single crystal Ag(111) substrate was prepared through cyclic sputtering (Ar^+, 10 min) and annealing (730 K, 15 min). The temperature of sample was measured by a thermocouple and a pyrometer. Germanium atoms were deposited on the Ag(111) surface with an electron beam evaporator (SPECS GmbH). A STM tip was made from the chemically etched tungsten. For constant height dI/dV imaging, the tip apex was terminated by a CO molecule picked up from the surface. The bias voltage was set close to zero voltage. The modulation amplitude was 7 mV_{rms} and the frequency was 510 Hz.

Theoretical calculations: Structural relaxations were realized with the VASP code,[1,2] implementing the PBE functional including the DFT-D3[3,4] method with Beck-Johnson damping function to account for possible vdW forces. Density of states, work function and Bader charge analyses shown in the main text were calculated with the HSE06 functional,[5-7] which considers a portion of exact exchange.[8] A plane-wave cutoff of 400 eV was used in all calculations. For PBE calculations, the GeNR was relaxed on the Ag(111) substrate with five layers using 0.01 eV/Å as the force tolerance. The two bottom layers of the Ag(111) substrate were kept fixed in their bulk position, and the k-grid sampling was set as 4x2x1. No further relaxation was done with the HSE06 functional and only the Γ-point was considered, with the two bottom Ag layers that were kept fixed in the relaxation with PBE removed from the structure. Density of states, work function and Bader charge analyses calculated with the PBE functional are shown in Figure S7.

For the STM simulations, we performed a single self-consistent loop in the all-electron code FHI-aims[9,10] using the geometry obtained with VASP. The "light" basis set was used with the PBE functional, and the unit cell was increased accordingly so the calculation could be realized with only the Γ-point. The two bottom Ag layers that were kept fixed in the relaxation were removed from the structure. The data generated with the FHI-aims code was then used to perform the STM simulations with the PP-STM code.[11,12] The CO tip was simulated by considering 13% and 87% tunnelling from s and p_{xy}-channels, respectively. The

electronic and atomic structures were visualized using Vesta.[13]

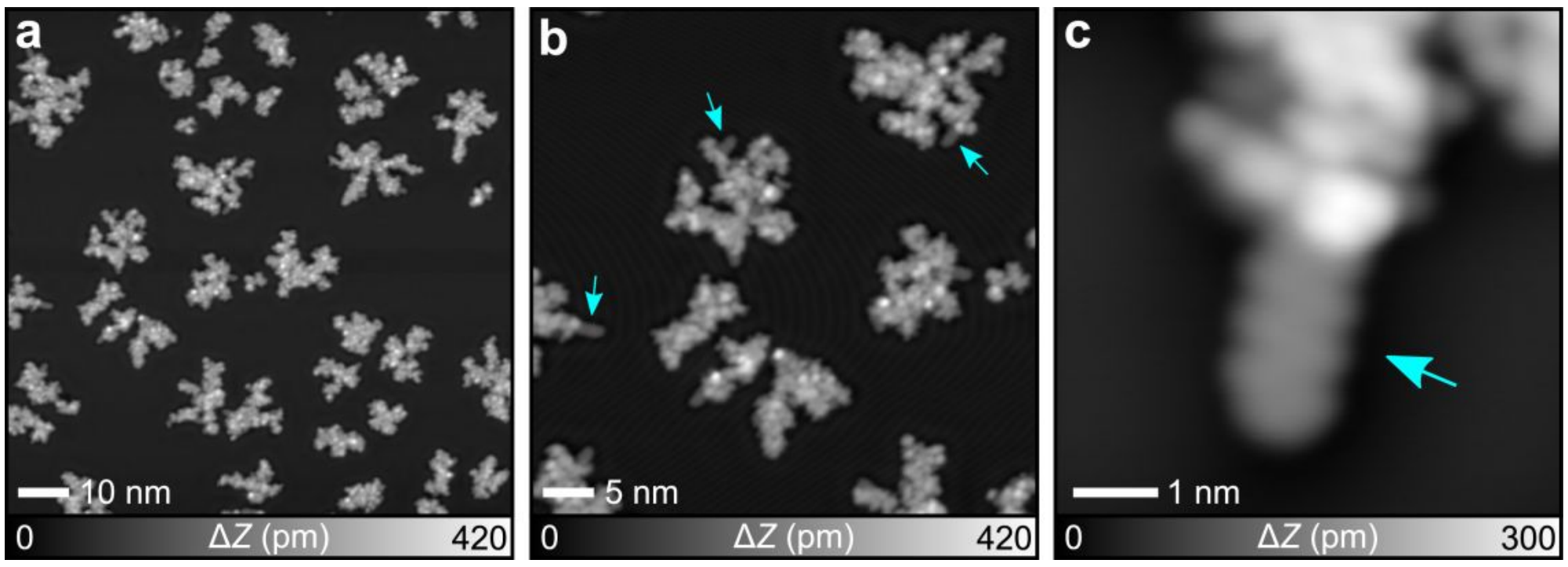

Figure S1. **As-deposited Ge atoms on Ag(111) held at 150 K.** (**a**,**b**) Large-scale STM topographies and (**c**) zoom-in STM topography. Very short GeNRs are occasionally observed, as indicated by the arrows. Measurement parameters: V = 200 mV and I = 5 pA in (a). V = 200 mV and I = 10 pA in (b). V = 10 mV and I = 10 pA in (c).

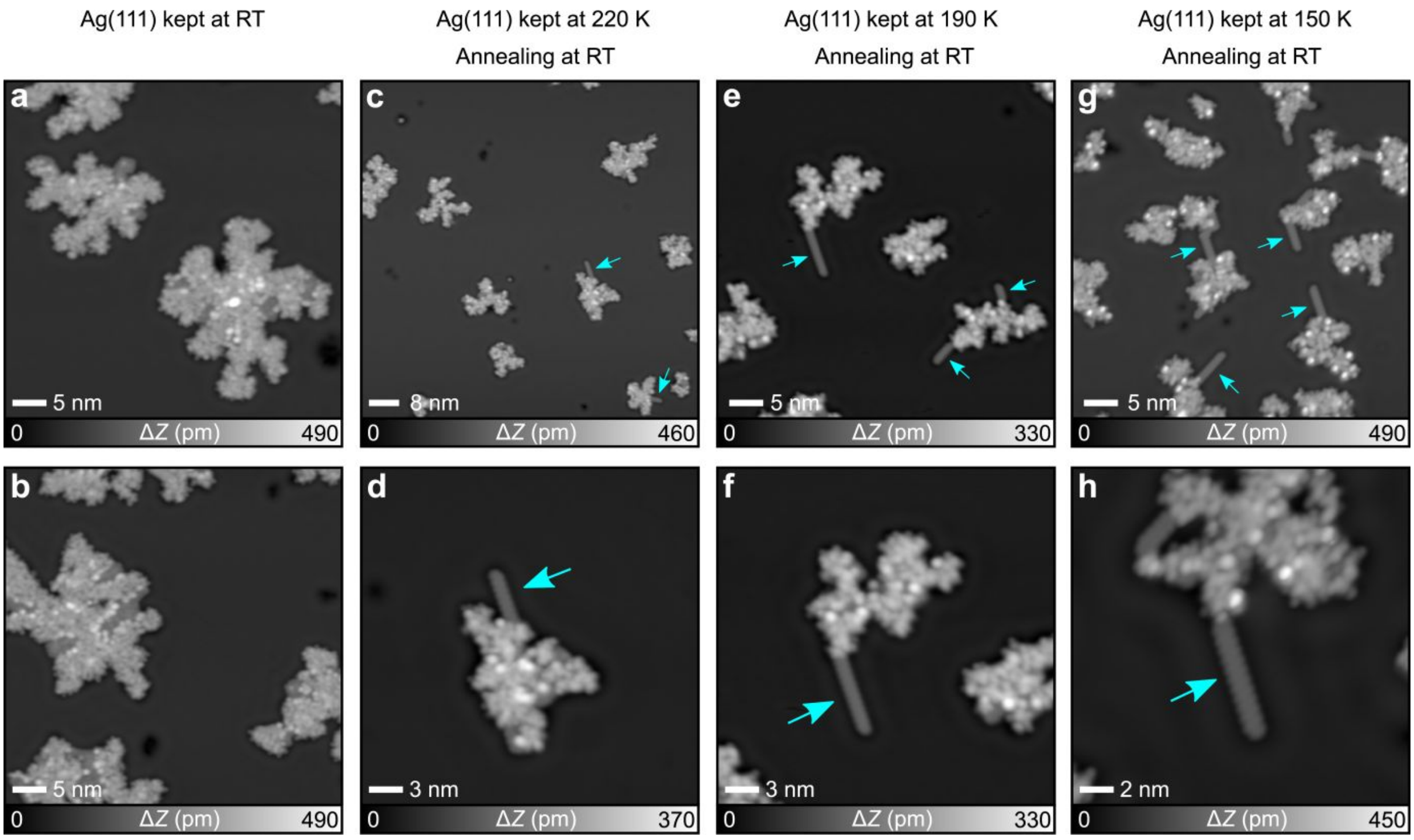

Figure S2. **Growth of GeNRs on Ag(111).** (**a**) STM topography of as-deposited Ge on Ag(111) held at room temperature (RT), and (**b**) the sample after keeping at RT for 1 h. STM topographies of Ge deposited at substrate temperatures of (**c**,**d**) 220 K, (**e**,**f**) 190 K and (**g**,**h**)150 K, each followed by RT annealing for 1 h. GeNRs are observed, as indicated by the arrows. Measurement parameters: V = 200 mV and I = 5 pA in (a),(b),(c),(d). V = 200 mV and I = 10 pA in (e),(f),(g). V = 100 mV and I = 10 pA in (h).

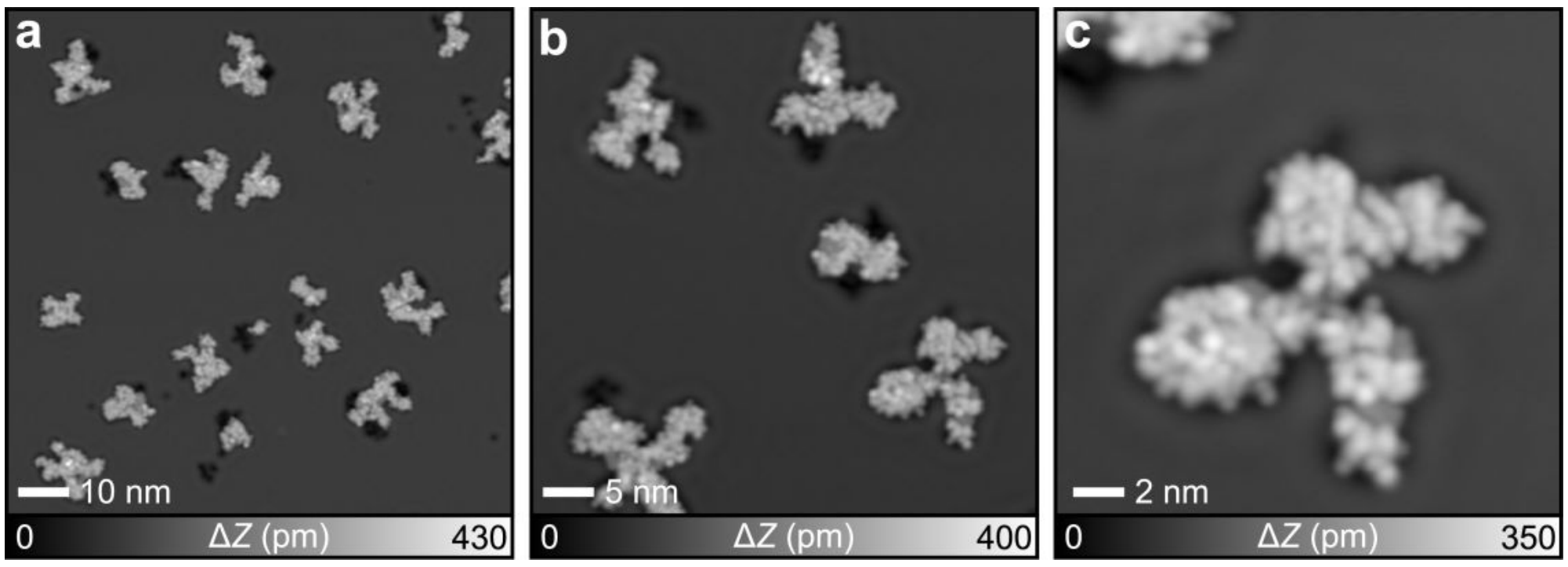

Figure S3. **Thermal stability test at 320 K.** (**a-c**) STM topographies of the sample after annealing at 320 K. Measurement parameters: V = 200 mV and I = 5 pA in (a). V = 200 mV and I = 10 pA in (b), (c).

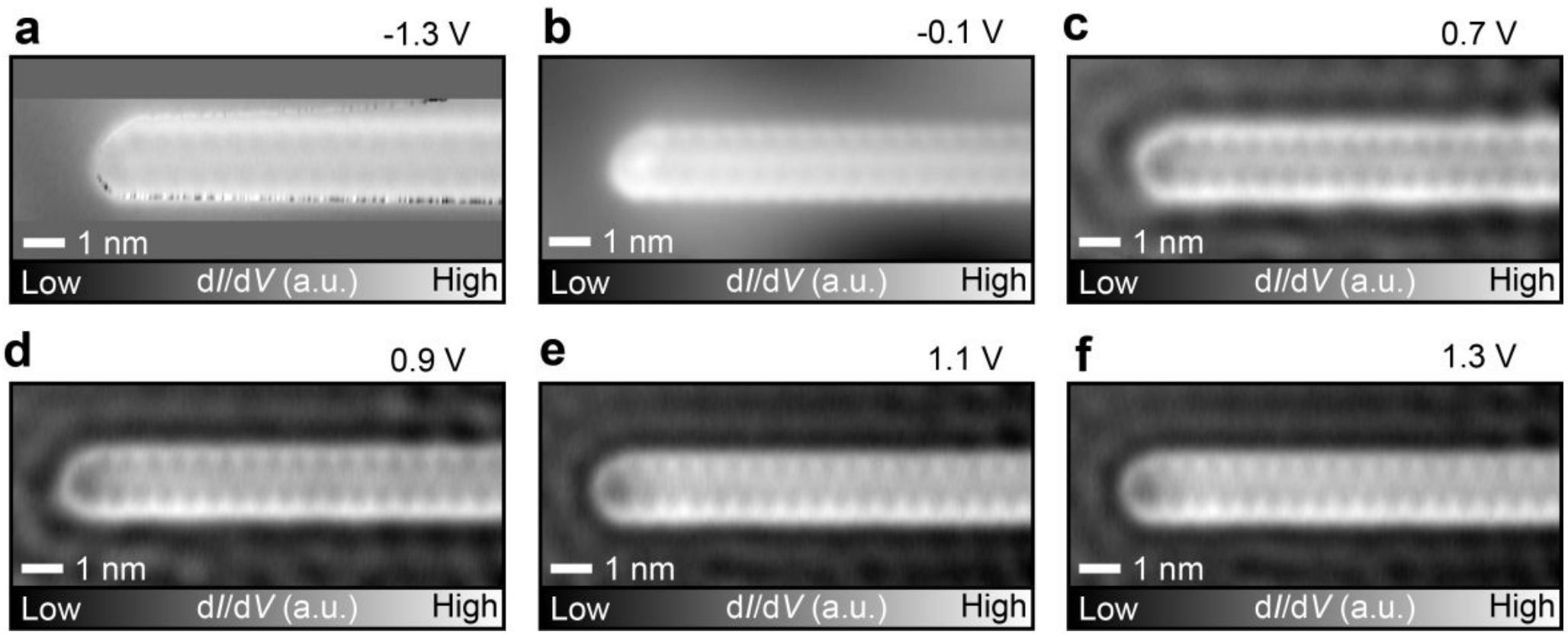

Figure S4. **Electronic properties of GeNR on Ag(111).** (**a**-**f**) Constant current d*I*/d*V* maps taken at: (a) –1.3 V, (b) –0.1 V, (c) 0.7 V, (d) 0.9 V, (e) 1.1 V, and (f) 1.3 V.

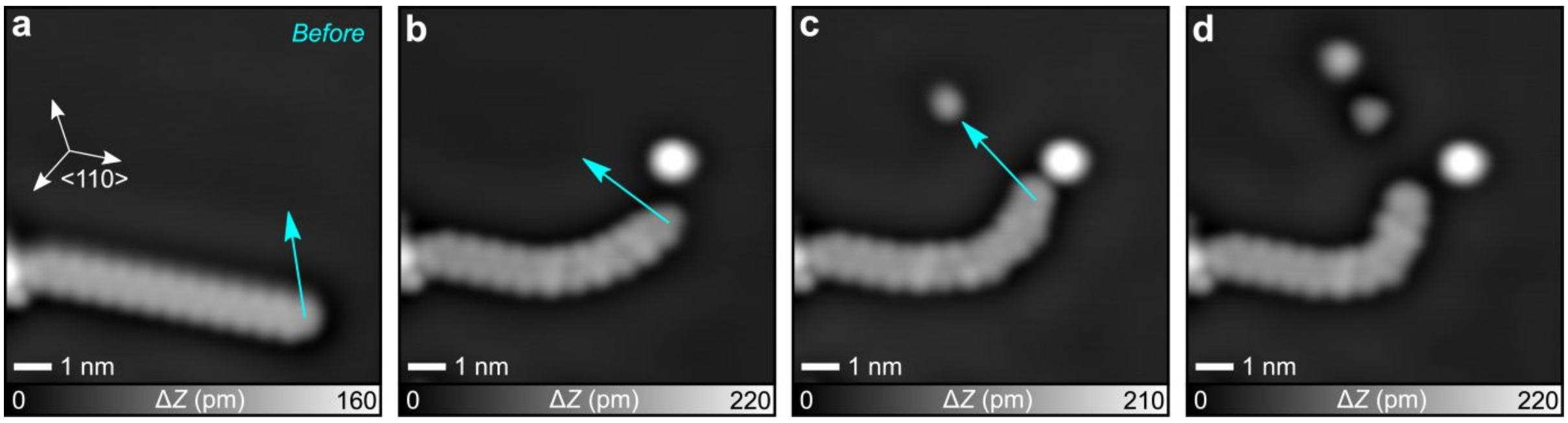

Figure S5. Tip-induced manipulation of GeNRs on Ag(111). Tip-assisted lateral movement of an individual GeNR: (**a**) before and (**b-d**) following a series of manipulations. Arrows indicate the tip movement direction. Measurement parameters: V = 200 mV and I = 10 pA.

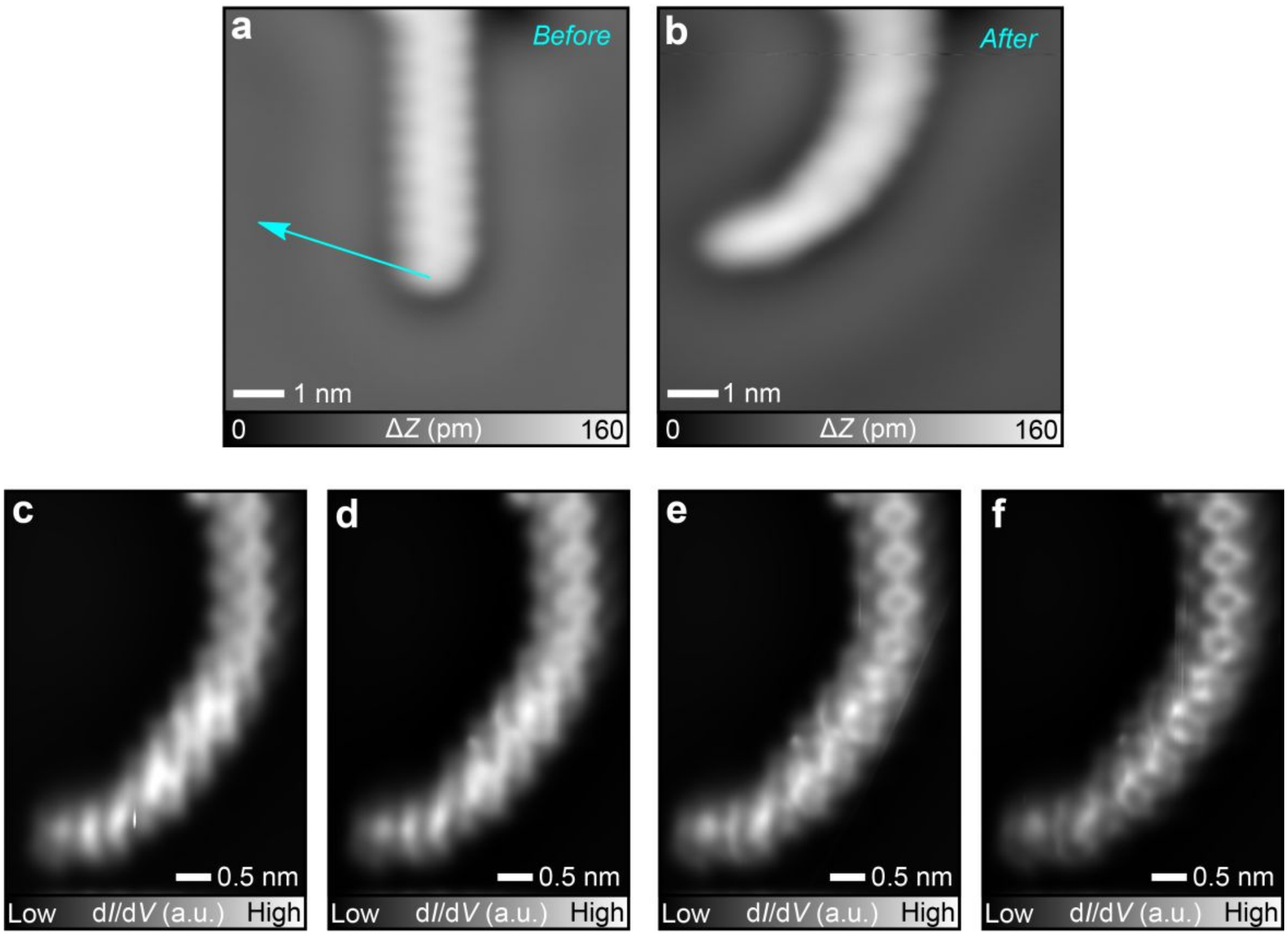

Figure S6. **Tip-induced manipulation of GeNRs on Ag(111).** Tip-assisted lateral movement of an individual GeNR: (**a**) before and (**b**) after. (**c**-**f**) A series of constant height dI/dV maps recorded with a CO-functionalized tip at reduced tip-surface separations. Measurement parameters: V = 200 mV and I = 10 pA in (a). V = 100 mV and I = 10 pA in (b).

Comparison between HSE06 and PBE functionals

In **Figure S7** is shown a very similar plot to **Figure 2** in the main manuscript, but with the properties calculated with the PBE (DFT-D3) functional, as opposed to the results calculated with the HSE06 functional (the experimental figure is the same as the one in **Figure 2a**). Overall, the PBE calculated PDOS is very similar to the HSE06 one, especially the behavior of the perpendicular p_z orbitals. A smaller gap is obtained in the p_x+p_y PDOS, but this is as expected. Regarding the Bader charge in **Figure S8,** the two-coordinated Ge atoms at the edges are more positive (losing 0.28e), with respect to HSE06 (losing 0.15e). The biggest discrepancy lies in the Bader charge within the GeNR, where a single Ge atom is slightly negative (gaining 0.03e), but the remaining atoms are all positive, in total contrast to the HSE06 values, where they are all either neutral or negative. This discrepancy is also reflected in the PBE calculated work function, which is lowered from 4.44 eV for bare Ag to 4.33 eV for GeNR+Ag).

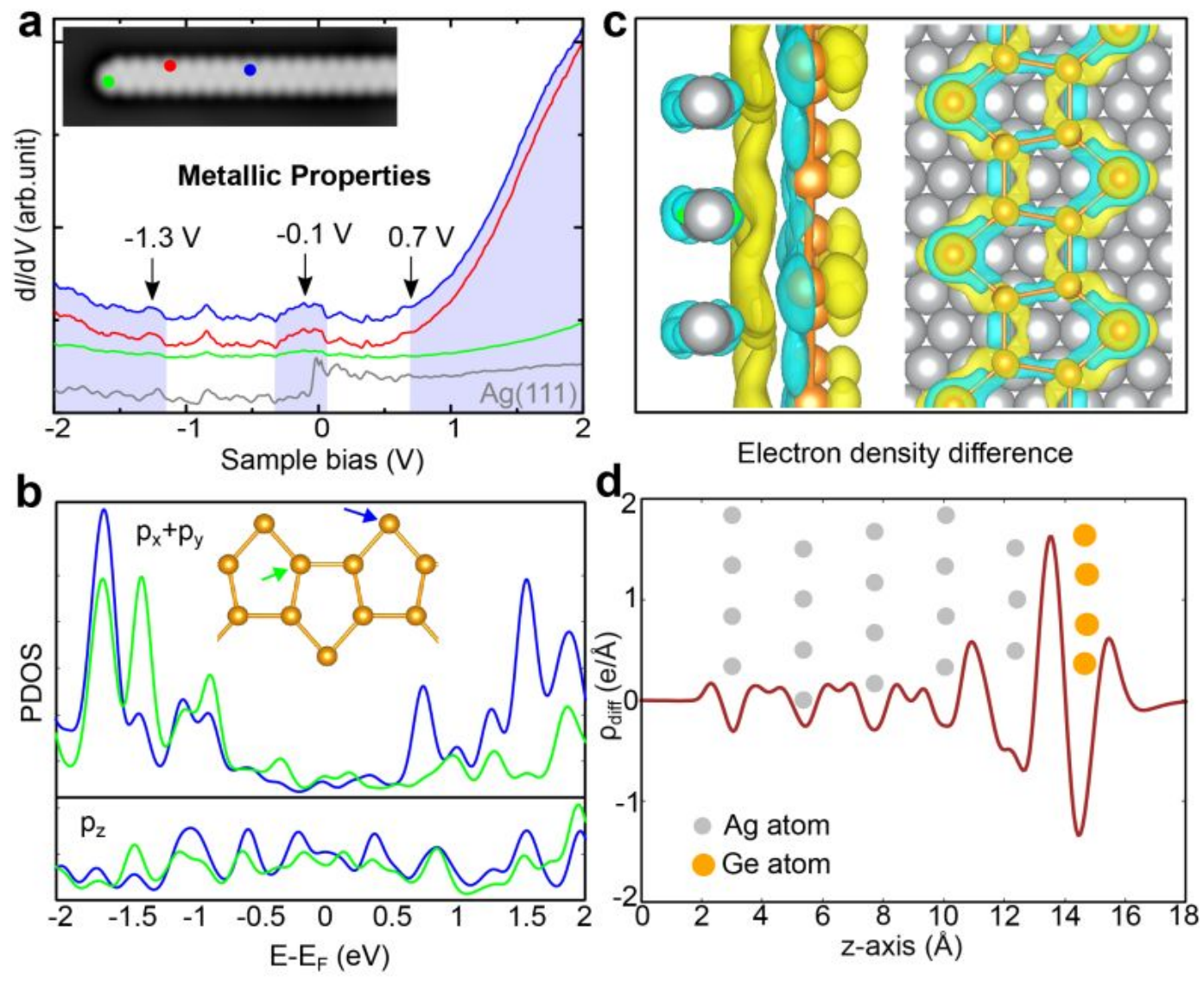

Figure S7. **Electronic properties of GeNR on Ag(111) calculated with PBE functional.** (**a**) d*I*/d*V* spectra recorded at different sites over the GeNR (indicated by color dots in the inset) and the bare Ag(111) surface. (**b**) Calculated DOS. (**c**) Side and top view of the DFT calculated electron density difference (±0.003 charge contours). Yellow isosurface reflects charge accumulation, while blue reflects depletion. (**d**) Planar average charge density difference plot, with the circles depicting the side view of the GeNR (in orange) on Ag(111) (in silver).

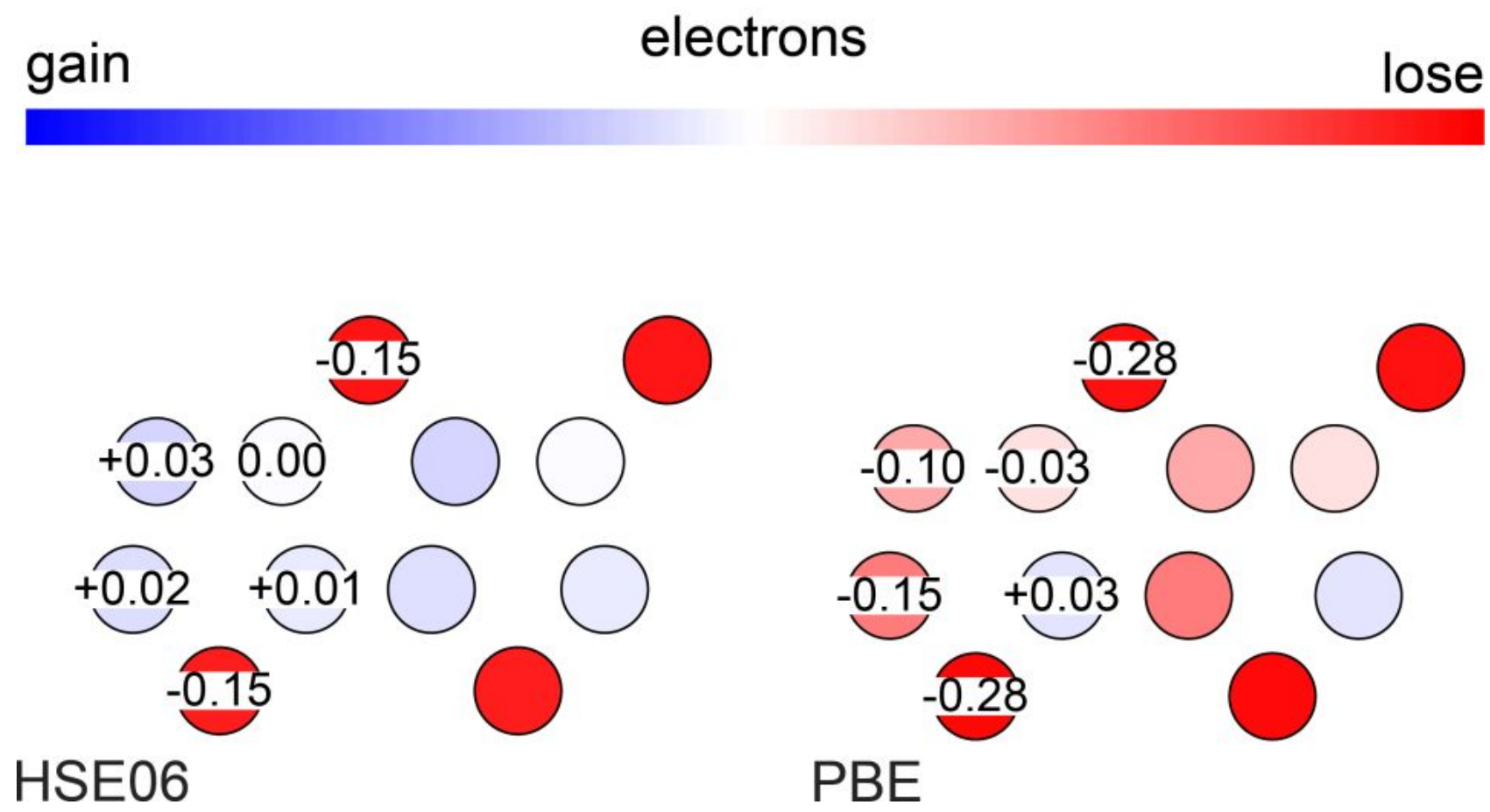

Figure S8. Bader charges of the inequivalent Ge atoms in the GeNR. The image shows the Bader charges calculated with both HSE06 and PBE functionals. The exact Bader charge is shown for the inequivalent atoms, calculated relatively to a neutral Ge atom (4 valence electrons). The color plot illustrates which atoms gain (blue) or lose electrons (red), highlighting the discrepancy between both functionals, especially for the three coordinated Ge atoms. The image also shows that the charge correlates to the height of the Ge atoms relatively to the substrate. Among the three coordinated Ge atoms, the ones that gained 0.03e and 0.02e are approximately 0.01 Å lower than the other two. The two coordinated Ge atoms are more than 0.1 Å lower than the three coordinated ones.

References

1. Kresse, G.; Furthmüller, J. Efficiency of ab-Initio Total Energy Calculations for Metals and Semiconductors Using a Plane-Wave Basis Set. *Comp. Mater. Sci.* **1996**, *6*, 15−50.
2. Kresse, G.; Furthmüller, J. Efficient Iterative Schemes for AB Initio Total-Energy Calculations Using a Plane-Wave Basis Set. *Phys. Rev. B* **1996**, *54*, 11169−11186.
3. Grimme, S.; Antony, J.; Ehrlich, S.; Krieg, S. A Consistent and Accurate AB Initio Parametrization of Density Functional Dispersion Correction (DFT-D) for the 94 Elements H-Pu. *J. Chem. Phys.* **2010**, *132*, 154104.
4. Grimme, S.; Ehrlich, S.; Goerigk, L. Effect of the Damping Function in Dispersion Corrected Density Functional Theory. *J. Comp. Chem.* **2011**, *32*, 1456−1465.

5. Heyd, J.; Scuseria, G. E. Efficient Hybrid Density Functional Calculations in Solids: Assessment of the Heyd−Scuseria−Ernzerhof Screened Coulomb Hybrid Functional. *J. Chem. Phys.* **2004**, *121*, 1187−1192.
6. Heyd, J.; Scuseria, G. E.; Ernzerhof, M. Hybrid Functionals Based on a Screened Coulomb Potential. *J. Chem. Phys.* **2003**, *118*, 8207−8215.
7. Heyd, J.; Scuseria, G. E.; Ernzerhof, M. Erratum: “Hybrid Functionals Based on a Screened Coulomb Potential” [J. Chem. Phys. 118, 8207 (2003)]. *J. Chem. Phys.* **2006**, *124*, 219906.
8. Perdew, J. P.; Burke, K.; Ernzerhof, M. Generalized Gradient Approximation Made Simple. *Phys. Rev. Lett.* **1996**, *77*, 3865−3868.
9. Havu, V.; Blum, V.; Havu, P.; Scheffler, M. Efficient O(N) Integration for All-Electron Electronic Structure Calculation Using Numeric Basis Functions. *J. Comput. Phys.* **2009**, *228*, 8367−8379.
10. Blum, V.; Gehrke, R.; Hanke, F.; Havu, P.; Havu, V.; Ren, X.; Reuter, K.; Scheffler, M. Ab Initio Molecular Simulations with Numeric Atom-Centered Orbitals. *Comput. Phys. Commun.* **2009**, *180*, 2175−2196.
11. Krejčí, O.; Hapala, P.; Ondràček, M.; Jelínek, P. Principles and Simulations of High-Resolution STM Imaging with a Flexible Tip Apex. *Phys. Rev. B* **2017**, *95*, 045407.
12. Oinonen, N.; Yakutovich, A. V.; Gallardo, A.; Ondráček, M.; Hapala, P.; Krejčí, O. Advancing Scanning Probe Microscopy Simulations: A Decade of Development in Probe-Particle Models. *Comput. Phys. Commun.* **2024**, *305*, 109341.
13. Momma, K.; Izumi, F. VESTA 3 for Three-Dimensional Visualization of Crystal, Volumetric and Morphology Data. *J. Appl. Cryst.* **2011**, *44*, 1272−1276.
